# Co-Chaperone Bag-1 Plays a Role in the Autophagy-Dependent Cell Survival through Beclin 1 Interaction

**DOI:** 10.3390/molecules26040854

**Published:** 2021-02-06

**Authors:** Miray Turk, Ozge Tatli, Hamza Furkan Alkan, Pelin Ozfiliz Kilbas, Gizem Alkurt, Gizem Dinler Doganay

**Affiliations:** 1 Molecular Biology, Genetics-Biotechnology, Graduate School of Science, Engineering and Technology, Istanbul Technical University, 34469 Istanbul, Turkey; turkm16@itu.edu.tr (M.T.); tatlio@itu.edu.tr (O.T.); alkurt.gizem@gmail.com (G.A.); 2 Department of Molecular Biology and Genetics, Istanbul Medeniyet University, 34720 Istanbul, Turkey; 3 Department of Molecular Biology and Genetics, Istanbul Technical University, 34469 Istanbul, Turkey; hfalkan@mit.edu; 4 Department of Molecular Biology and Genetics, Istanbul Kultur University, 34140 Istanbul, Turkey; p.ozfiliz@iku.edu.tr

**Keywords:** Bag-1, Bcl-2, Beclin 1, autophagy, breast cancer

## Abstract

Expression levels of the major mammalian autophagy regulator Beclin 1 and its interaction with Bcl-2 regulate the switch between autophagic cell survival and apoptotic cell death pathways. However, some of the regulators and the precise mechanisms of these processes still remain elusive. Bag-1 (Bcl-2 associated athanogene-1), a member of BAG family proteins, is a multifunctional pro-survival molecule that possesses critical functions in vital cellular pathways. Herein, we report the role of Bag-1 on Bcl-2/Beclin 1 crosstalk through indirectly interacting with Beclin 1. Pull-down experiments suggested a molecular interaction between Bag-1 and Beclin 1 in breast cancer cell lines. On the other hand, in vitro binding assays showed that Bag-1/Beclin 1 interaction does not occur directly but occurs through a mediator molecule. Bag-1 interaction with p-Beclin 1 (T119), indicator of early autophagy, is increased during nutrient starvation suggesting involvement of Bag-1 in the autophagic regulation. Furthermore, CRISPR/Cas9-mediated Bag-1 knock-out in MCF-7 cells hampered cell survival and proliferation and resulted in decreased levels of total LC3 under starvation. Collectively, we suggest that Bag-1 modulates cell survival/death decision through maintaining macroautophagy as a component of Beclin 1-associated complexes.

## 1. Introduction

In mammals, two major degradation pathways exist to restore protein homeostasis, also referred as proteostasis: (1) the ubiquitin-proteasome system (UPS), which is responsible for the degradation of the most short-lived, abnormal or denatured proteins; and (2) autophagy, which is responsible for the degradation of aggregates and cellular organelles. The simultaneous action of both systems ensures the precise control of transcription, DNA repair, cell stress response, and apoptosis, and thus modulates cell death and survival decisions in the cell [1,2,3].

Chaperones and co-chaperones constitute the first line of proteostasis by maintaining a correctly folded proteome. The Bcl-2 associated anthanogene (BAG) family is a group of co-chaperones (from Bag-1 to Bag-6) each sharing an evolutionary conserved BAG domain at their C-terminus which allows their interaction with Heat Shock Protein 70 kDa (Hsp70/Hsc70) [4,5,6]. Co-chaperone Bag-1 functions as a nucleotide exchange factor (NEF) for the chaperone Hsp70 and triggers the release of chaperone-bound clients [7,8]. It also participates in the switch between proteasomal degradation pathway and autophagy to tune proteostasis [9,10,11]. Central ubiquitin-like (UBL) domain of Bag-1 provides direct interaction with 26S proteasome [8].

Bag-1 is a multifunctional protein and is produced as three major isoforms through alternative translation initiation. These three isoforms, each with a distinct N-terminus, include Bag-1S (~33 kDa), Bag-1M (~46 kDa), and Bag-1L (~52 kDa) [12]. Bag-1L, which possesses a nuclear localization signal, is mainly a nuclear protein, whereas Bag-1M partitions between nucleus and cytoplasm [13]. Bag-1S is translated through an internal ribosome entry segment (IRES)-mediated initiation [14], lacks the nuclear localization signal, and therefore is predominantly found in the cytoplasm [15]. Bag-1 has a diverse array of interaction partners like Bcl-2 (B-cell lymphoma 2), Hsp70/Hsc70, nuclear hormone receptors, including estrogen, glucocorticoid or thyroid receptors, and members of the ubiquitylation mechanism [12,13]. Both nuclear and cytoplasmic Bag-1 has been implicated in cell survival mechanisms [15,16,17]. Bag-1 can reduce the caspase activation and apoptosis initiation in human cervical multidrug-resistant cells [9,18] and can also potentiate nuclear hormone receptors, which are recognized as major regulators of proliferation and survival [19,20]. Bag-1 overexpression upregulates one of the main apoptosis regulator Bcl-2 in Jurkat cells [19,21], while its downregulation decreases Bcl-2 levels in cervical cells [22]. The formation of Bag-1/Bcl-2 complex activates the Ras/Raf/MEK/ERK pathway, which controls the transcription of cell survival genes [23,24]. Hence, Bag-1 functions as an anti-apoptotic protein involved in both activation of cell survival and inhibition of cell death through a variety of mechanisms [12,19,22,25].

Bcl-2 is a major anti-apoptotic protein and is elevated in about 75% of primary breast tumors from human patients [26]. It functions through the formation of homodimers and heterodimers, especially with Bag-1, Bax, Bad, Bak, Beclin 1 and Raf-1 [22,24,25]. The association of Bcl-2 with the autophagy regulator Beclin 1 modulates the switch between apoptosis and autophagy pathways [27,28]. Bcl-2/Beclin 1 interaction is regulated by competitive binding, self-association, or post-translational modifications of Beclin 1 or Bcl-2 [29]. Death-associated protein kinase (DAPK), Erk, and JNK contribute to this regulation through a series of phosphorylation events [26,27,28,29,30,31,32]. Understanding these important nodes in the crosstalk between autophagy and apoptosis is crucial for the improvement of cancer therapies.

Autophagy is subdivided into macroautophagy, microautophagy and chaperone-mediated autophagy. During macroautophagy, molecules to be degraded are enveloped in autophagosomes, which will then be transported and fused with lysosomes to form autolysosomes. During microautophagy, target molecules are basically engulfed by the lysosomal membrane for degradation. Microautophagy may target various cytoplasmic proteins for diverse purposes [33]. Lastly, in chaperone-mediated autophagy (CMA), chaperones recognize clients and recruit them directly to lysosomal membrane [3,26,34,35,36]. BAG family co-chaperones, like Bag-3, were previously reported as associated with chaperone-mediated autophagy (CMA), which does not require the formation of autophagosomal bodies [27,37]. In addition, Bag-1 sustains protein homeostasis through supporting proteasomal degradation [33,38,39]. Our current work suggests that Bag-1 may have another function to maintain protein homeostasis through supporting autophagosome-mediated macroautophagy via Beclin 1 interaction.

In this study, we reported Bag-1 as a potential regulator of autophagic cell survival through Beclin 1 interaction. Our data suggested that Bag-1 indirectly forms a complex with Beclin 1 through at least one bridging molecule and improves autophagy and cell survival during starvation in breast cancer cell lines. Further studies may reveal Bag-1/Beclin 1 complex as a targetable component of autophagic progress in breast carcinomas. 

## 2. Results

### 2.1. Bag-1 Interacts with Beclin 1

**Figure 1:**
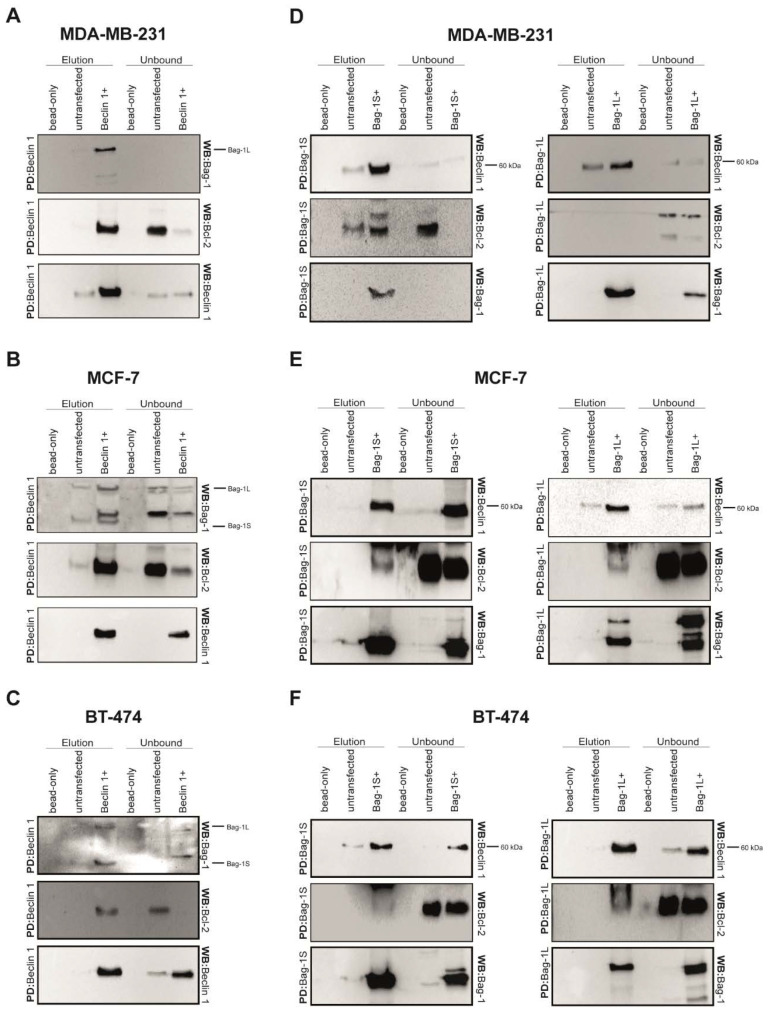
Beclin 1 interacts with both Bag-1S and Bag-1L isoforms. (**A**–**C**) His_6_Beclin 1 was his-pulled down by Ni-NTA beads from (**A**) MDA-MB-231, (**B**) MCF-7, and (**C**) BT-474 cell lysates. After wash, bound Beclin 1-related complexes were released by imidazole. As a control, precipitations were also carried out in parallel experiments with untransfected cell lysate and empty Ni-NTA beads. Elution and unbound fractions were subjected to western blot analysis using anti-Bag-1, anti-Bcl-2, anti- Beclin 1 antibodies. (**D**–**F**) Both His_6_Bag-1S and His_6_Bag-1L were his-pulled down in (**D**) MDA-MB-231, (**E**) MCF-7 and (**F**) BT-474 cells as reciprocal experiments and analyzed by western blotting. Bag-1 and Beclin 1 were co-precipitated in all experiments.

Known interactions of Bcl-2 with both Beclin 1 [9,27,40,41,42] and Bag-1 has prompted us to search for a possible Bag-1/Beclin 1 complex. We hypothesized that Bag-1 might have a function in macroautophagic regulation by interacting with Beclin 1. To test this hypothesis, we independently overexpressed N-terminally His_6_-tagged Bag-1S (His_6_Bag-1S), Bag-1L (His_6_Bag-1L), and Beclin 1 (His_6_Beclin 1) proteins in MDA-MB-231, MCF-7, and BT-474 human breast cancer cell lines since they exhibit differences in their prognosis to the disease progression due to their origin and the rates of survival and recurrence. MCF-7 cells are noninvasive, positive for both estrogen receptor (ER+) and progesterone receptor (PR+) and lack the human epidermal growth factor receptor (HER-2), whereas MDA-MB-231 and BT-474 are triple-negative and triple-positive breast cancer cell lines. In hormone-receptor positive, HER2 negative cancer cells, the lack of HER2 makes them unsusceptible to Trastuzumab [43]. Endocrine therapy is the first line treatment of choice; however, patients suffer from acquired resistance to the therapy. To improve survival, it is sufficient to seek new therapeutic targets and modalities. For that purpose, we focused on MCF-7 cells to study the effect of Bag-1 on autophagic regulation through an interaction with Beclin 1. His_6_-Bag-1S, His_6_-Bag-1L or His_6_Beclin 1-related protein complexes were isolated using affinity-based Ni-NTA agarose beads under native conditions and analyzed by immunoblotting. Pull-down experiments demonstrated that endogenous Bag-1 binds to immobilized His_6_Beclin 1 in all three cell lines but does not bind to control matrices (Figure 1A–C, Figure S1). Bag-1M was not detected in any of the cell lines and Bag-1S was not found in MDA-MB-231 cell line. Reciprocally, endogenous Beclin 1 was also found to associate with His_6_Bag-1S and His_6_Bag-1L in the three cell lines. Unexpectedly, although Bcl-2 is one of the most widely known interaction partner of Bag-1, only very little amount of Bcl-2 co-precipitated with Bag-1 (Figure 1D–F, Figure S1). Regardless, these findings imply that Bag-1 forms a complex with Beclin 1 and therefore may contribute to the autophagic regulation.

### 2.2. Bag-1S Isoform, but Not Bag-1L Isoform Intracellularly Co-Localizes with Beclin 1

**Figure 2:**
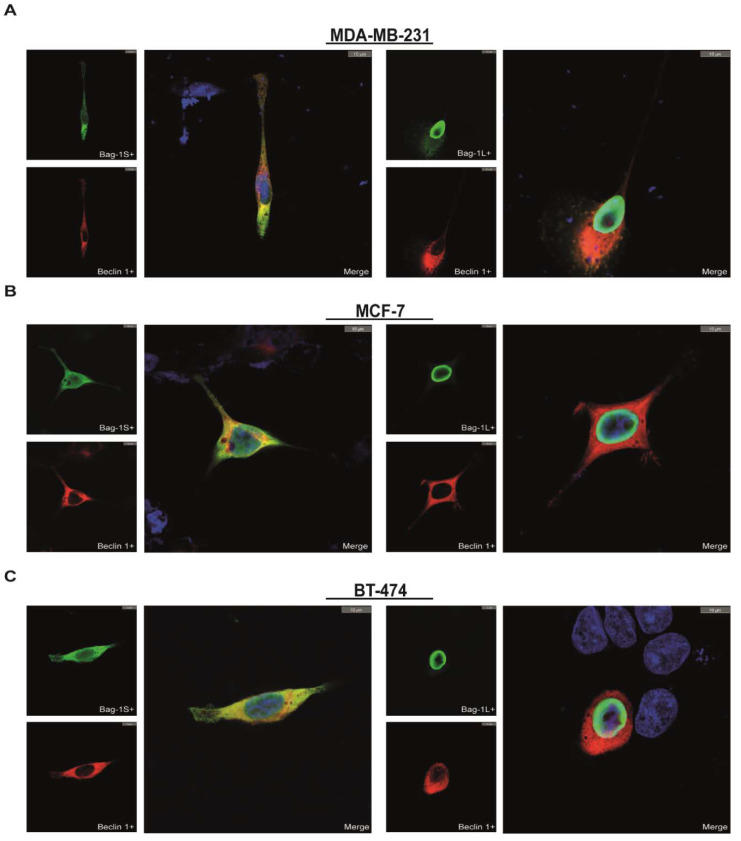
Interaction of Bag-1S and Beclin 1 is revealed in situ by immunocytochemistry assay. (**A**) MDA-MB-231, (**B**) MCF-7, and (**C**) BT-474 cells were co-transfected with His_6_Beclin 1 and His_6_Bag-1S or His_6_Bag-1L and their co-localization is investigated via immunocytochemistry. Bag-1S and Bag-1L is independently stained green and Beclin 1 was stained as red while nucleus was shown in blue. Co-localized areas were represented in yellow. Scale bar equals to 10 µm. In all cell lines, Bag-1S and Beclin 1 was observed in cytoplasm while Bag-1L is located inner side of nuclear membrane. Only small isoform of Bag-1 is observed co-localized to Beclin 1.

All Bag-1 isoforms have both BAG and ULD domains, however they differ based on their cellular localizations. Although His_6_-pull down experiments showed that Beclin 1 interacts with both small and large isoforms of Bag-1 (Figure 1), these interactions may occur artificially throughout the cell lysis or bead-binding stage of pull-down processes. To test whether these interactions are biologically relevant, subcellular localizations of Bag-1 isoforms and Beclin 1 were analyzed in situ by immunocytochemistry. His_6_Beclin 1 and His_6_Bag-1S or His_6_Bag-1L were co-expressed in MDA-MB-231, MCF-7, and BT-474 cells and localizations of Beclin 1 and Bag-1 were determined. Bag-1S was homogeneously dispersed in the cytosol, while Bag-1L was predominantly located at the inner side of the nucleus (Figure 2, Figure S2). Beclin 1 was also localized mostly in the cytosol, while only a very small fraction was detected in the nucleus (Figure 2). These data suggest that Bag-1L only interacts with Beclin 1 throughout the cell lysis or pull down processes due to structural similarity to Bag-1S but they do not interact endogenously as they are located in different cellular compartments. Therefore, we hypothesize that only Beclin 1/Bag-1S interaction, but not Beclin 1/Bag-1L interaction occurs endogenously and is potentially relevant for biological events including autophagy.

### 2.3. Bag-1/Beclin 1 Interaction Occurs before Induction of Autophagy

**Figure 3:**
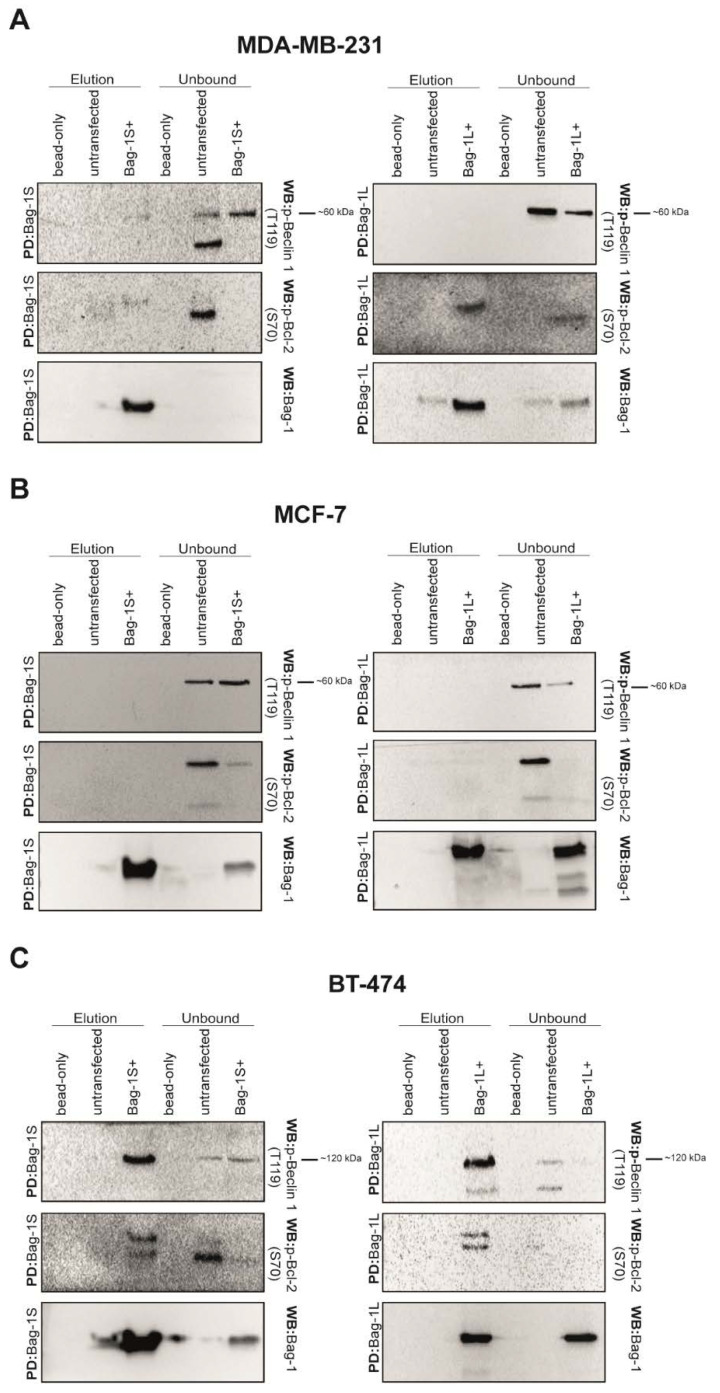
p-Beclin 1 (T119) or p-Bcl-2 (S70) do not form a complex with Bag-1 isoforms in MDA-MB-231 and MCF-7 cells. Both Bag-1S and Bag-1L isoforms were precipitated with Ni-NTA agarose beads from MDA-MB-231 (**A**), MCF-7 (**B**) and BT-474 (**C**) cell lysates. As a control, untransfected cell lysate and empty Ni-NTA beads were subjected to Ni-NTA precipitation in parallel experiments. Elution and unbound fractions were analyzed by western blotting using anti-Bag-1, anti-Bcl-2 (S70), anti- Beclin 1 (T119) antibodies. P-Beclin 1 (T119) was not found in Bag-1 precipitates in MDA-MB-231 and MCF-7 cells.

Next, p-Beclin 1 (T119) and p-Bcl-2 (S70), indicators of Beclin 1-mediated autophagy initiation [28], were also examined in both His_6_Bag-1S and His_6_Bag-1L precipitates and neither p-Beclin 1 (T119) nor p-Bcl-2 (S70) was detected in the same complex with Bag-1 in MDA-MB-231 (Figure 3A) or MCF-7 (Figure 3B) cell lines. On the other hand, an intact p-Beclin 1 (T119) band corresponding to ~120 kDa was detected in Bag-1S and Bag-1L precipitates in BT-474 cell line (Figure 3C), which can be speculated that Bag-1 interacts with p-Beclin 1 (T119) dimer. Together, these data suggest that Bag-1 could interact with Beclin 1 before the phosphorylation-dependent sequestration of Beclin 1 from Beclin 1/Bcl-2 assembly although some variations are observed among different cell lines (Figure 3).

**Figure 4:**
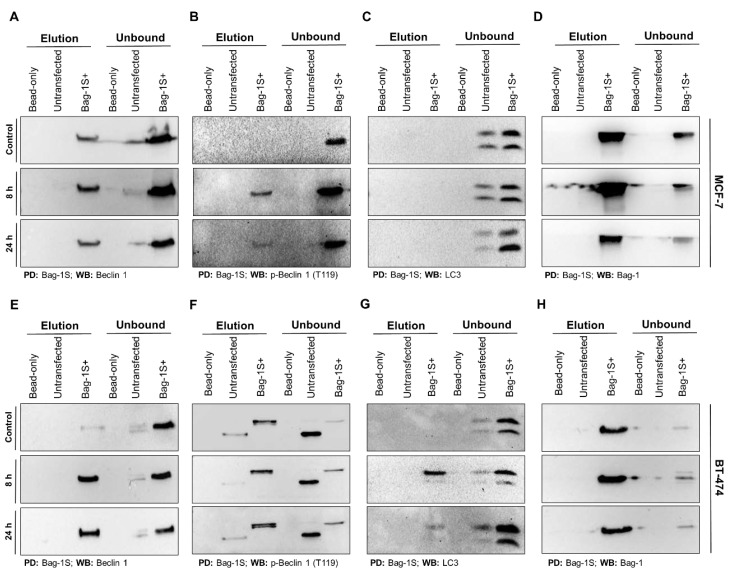
Bag-1S/Beclin 1 interaction occurs independent of autophagy. Bag-1S was overexpressed in MCF-7 and BT-474 cells and cells were starved for 8 h or 24 h in serum- and glutamine-free DMEM media for autophagy induction. Normal media condition was used as non-starved control. Total cell lysate was his-pulled down by Ni-NTA beads. After wash, Bag-1S bound protein complexes were eluted by imidazole. As negative control, precipitations were also carried out in parallel experiments with untransfected cell lysate and empty Ni-NTA beads. Elution and unbound fractions of MCF-7 and BT-474 cell samples were subjected to western blot analysis using anti- Beclin 1 (**A**,**E**), anti-p-Beclin 1 (T119) (**B**,**F**), and anti-LC3 antibodies (**C**,**G**), respectively. LC3-II/I ratio of unbound fraction belonging to Bag-1S+ cells were used as a control for autophagy induction. Detection of Bag-1 through immunoblotting is performed for precipitation control (**D**,**H**). PD: Pull-down; WB: western blot.

During the early stages of autophagy, Beclin 1 is phosphorylated (T119) and is dissociated from Bcl-2 [44]. Because Bag-1S displayed higher binding affinity for Beclin 1 than p-Beclin 1, we hypothesized that Bag-1/Beclin 1 interaction might be regulated throughout autophagy process. To test this hypothesis, we pulled-down His_6_Bag-1S from serum- and glutamine-starved MCF-7 and BT-474 cells and examined Beclin 1, p-Beclin 1 (T119) and LC3. Interestingly, Bag-1S/Beclin 1 interaction was not apparently altered by starvation in MCF-7 cells (Figure 4A), while stronger interaction was observed in BT-474 under starvation condition, compared to non-starved condition (Figure 4E).

On the other hand, Bag-1S/p-Beclin 1 association was induced during starvation in MCF-7 cells (Figure 4B), while upon starvation in BT-474 cells, this interaction remained at comparable levels to those observed in control media conditions (Figure 4F). This suggests that Bag-1 shows lower affinity to p-Beclin 1 (T119) in standard media conditions in MCF-7 cells, but nutrient starvation increases the affinity of Bag-1 to p-Beclin 1. In contrast, Bag-1S and p-Beclin 1 co-precipitated independent of media condition in BT-474 cells. Also, a slightly higher molecular weight was detected for p-Beclin 1 in Bag-1S-overexpressed BT-474 cells compared to untransfected cells, which probably indicates the presence of post-translational modifications (Figure 4F). In addition, we also determined that LC3 protein does not interact with Bag-1S in MCF-7 cells regardless of media conditions (Figure 4C), while LC3 was co-precipitated with Bag-1S in BT-474 cells under starvation condition (Figure 4G). Importantly, levels of lipidated form of LC3 (LC3-II), important mediator of autophagosome biogenesis [45], was elevated upon starvation, indicating that serum and glutamine starvation induced autophagy. Precipitation control is performed through immunoblotting of Bag-1 in each pull down sample (Figure 4D,H).

### 2.4. Bag-1 Does Not Directly Interact with Beclin 1 In Vitro

**Figure 5:**
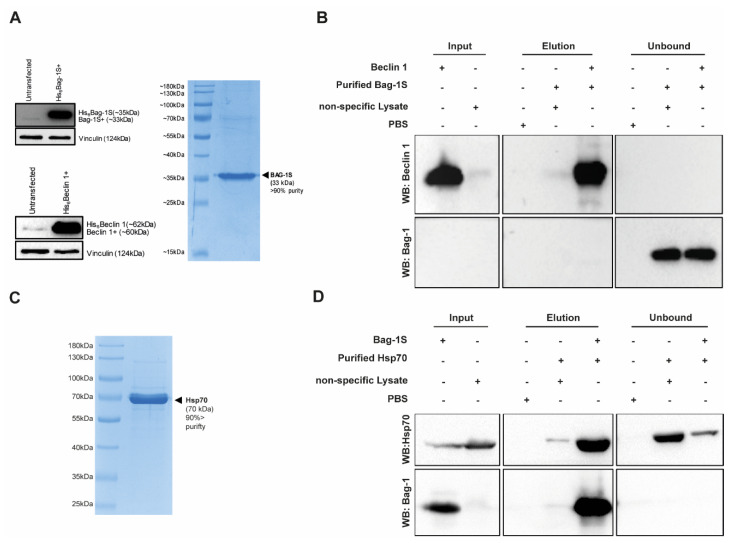
Bag-1S and Beclin 1 does not interact directly in vitro. (**A**) Full-length mammalian Bag-1S and Beclin 1 were overexpressed in HEK293T cells and Bag-1S was purified via two-step affinity purification (>90% purity). (**B**) His_6_-Beclin 1 was immobilized to Ni-NTA agarose beads and stringently washed. Purified Bag-1S and Ni-NTA-immobilized Beclin 1 was incubated at 37 °C with to allow formation of protein-protein interaction. Bound protein complexes were released by imidazole. PBS and non-specific cell lysates were used as negative control for Ni-NTA binding. Bag-1S and Beclin 1 were not eluted together. (**C**) Purity of Hsp70 is determined by SDS-PAGE. (**D**) His_6_-Bag-1S was immobilized to Ni-NTA agarose beads and stringently washed. Purified Hsp70 and Ni-NTA-immobilized Bag-1S was incubated at 37 °C with to allow formation of protein-protein interaction. Bound protein complexes were released by imidazole. PBS and non-specific cell lysates were used as negative control for Ni-NTA binding.

To explore whether Bag-1 binds to Beclin 1 directly, we designed an in vitro binding experiment. Firstly, we overexpressed His_6_Bag-1S and His_6_Beclin 1 in HEK293T cells which are easy to transfect and the level of protein expression is very high. Lysates from Bag-1S overexpressing cells were subjected to two-step affinity purification using Ni-NTA beads and Bag-1S was collected through flow-through mode (Figure 5A). Then, purified Bag-1S was incubated in vitro at 37 °C for 30 min with Ni-NTA-immobilized His_6_Beclin 1. As a negative control (Bag-1 only), we prepared a cell lysate from wild-type cells (without expressing His_6_Beclin 1) to eliminate false-positives resulting from non-specific interactions of the cell lysate with Ni-NTA beads. Also, we conducted a parallel experiment with His_6_Beclin 1 overexpressing cell lysate without incubation with Bag-1 as another control. Bag-1S and Beclin 1 did not interact with each other under these conditions and were detected in different fractions (Figure 5B). As a positive control for in vitro binding assay, we verified binding conditions through Bag-1S/Hsp70 interaction. To achieve this, purified Hsp70 (Figure 5C) was incubated in vitro with Ni-NTA-immobilized His_6_Bag-1S as performed for Bag-1S/Beclin 1 interaction. Upon incubation at 37 °C for 30 min, Hsp70 and Bag-1S were detected in the same fraction indicating that they do interact under the same in vitro binding conditions where Bag-1S and Beclin 1 failed to interact (Figure 5D). These experiments showed that His_6_Beclin 1 do not engage in a direct physical association with Bag-1S, at least under our experimental conditions (Figure 5). Since in vitro binding assay implied that there is no direct interaction between Bag-1S and Beclin 1, we speculated that the formation of a Bag-1/Beclin 1 complex might be mediated by an intermediary molecule.

### 2.5. Bag-1 Deficiency Impairs Cell Survival and Proliferation

**Figure 6:**
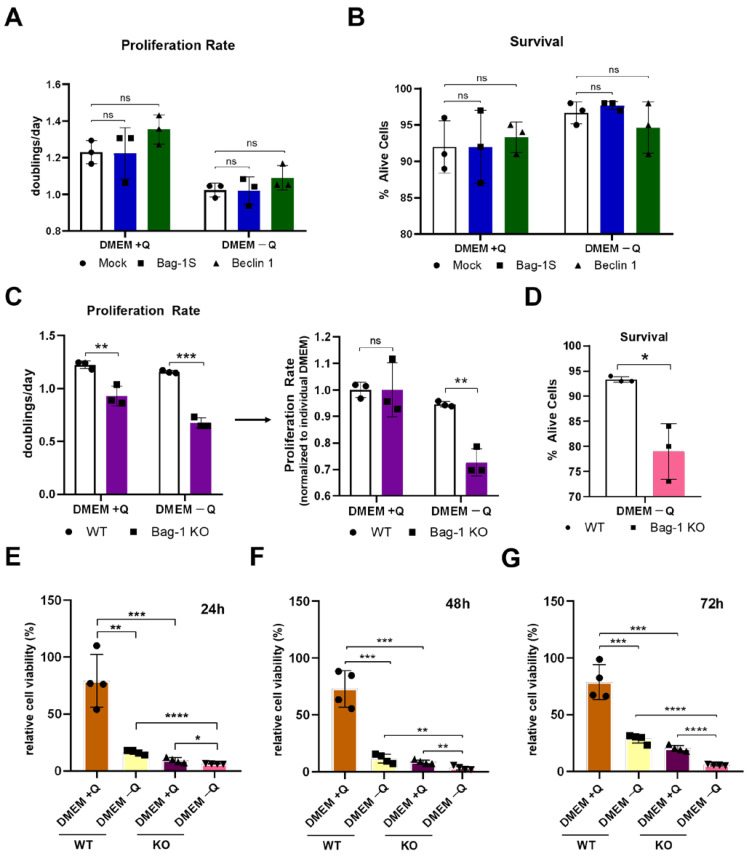
Bag-1 deficiency impairs cell proliferation and survival. (**A**) Proliferation rate of MCF-7 cells transfected with empty pcDNA 3.1 (Mock), Bag-1S, Beclin 1. Cells were incubated in DMEM with or without glutamine (Q) for three days, cells were counted via cell counter device, and proliferation rate was calculated as doubling/day. (**B**) Percent of cells from (A) that were trypan blue negative. (**C**) (Left) Proliferation rate of WT and Bag-1 knock-out (Bag-1 KO) cells treated in either standard DMEM or DMEM without glutamine (Q) for three days. (Right) Proliferation rates of the WT and Bag-1 KO cells in DMEM without glutamine were represented after normalization to corresponding proliferation rates in standard DMEM. Cells were counted and proliferation rates were calculated as doublings/day. (**D**) Percent of cells from (C) that were trypan blue negative. Wild-type and Bag-1 knock-out cells were grown in serum and glutamine containing DMEM (DMEM + Q) or serum and glutamine-free (DMEM − Q) media. MTT assay was performed upon 24 h (**E**), 48 h (**F**) or 72 h (**G**) starvation under specified conditions.

Because Bag-1S forms a complex with Beclin 1, we hypothesized that Bag-1S might play a role in cellular proliferation and survival processes. To test this, we transiently transfected MCF-7 cells with Bag-1S or Beclin 1 and performed a cell counting assay. Interestingly, Bag-1S overexpression did not significantly improve the proliferation compared to empty vector (Mock) (Figure 6A). To investigate whether Bag-1 would mediate proliferation when autophagy is induced, we depleted cells from glutamine for three days and measured proliferation. However, we did not observe any significant changes in neither proliferation rate (Figure 6A) nor cell survival (Figure 6B) between Bag-1S or Mock transfected MCF-7 cells upon glutamine starvation. In addition, Beclin 1 overexpression also did not significantly improve cell survival or proliferation regardless of the media conditions. These data suggest that overexpression of neither Bag-1S or Beclin 1 improves proliferation.

Next, we tested whether the lack of Bag-1 would impact proliferation. We performed cell proliferation assays on CRISPR/Cas9-mediated Bag-1 knock-out (Bag-1 KO) MCF-7 cells. Bag-1 KO cells proliferated dramatically slower than wild-type cells (WT), even in standard, serum and glutamine-containing media conditions (Figure 6C). Strikingly, this phenotype was further exacerbated by glutamine starvation (Figure 6C), suggesting that Bag-1 KO cells are particularly vulnerable to conditions in which active autophagic flux is required. Next, we investigated cell viability using trypan blue staining and MTT assay. Consistently, number of viable cells upon glutamine starvation was also reduced in Bag-1 KO cells compared to WT cells (Figure 6D–G). All data together reflected that increased expression of Bag-1 is redundant for proliferation and cell survival while lack of Bag-1 hampers proliferation and decreases cell viability, especially upon glutamine starvation. 

### 2.6. Bag-1 Participates in Autophagic Regulation in MCF-7 Cells

**Figure 7:**
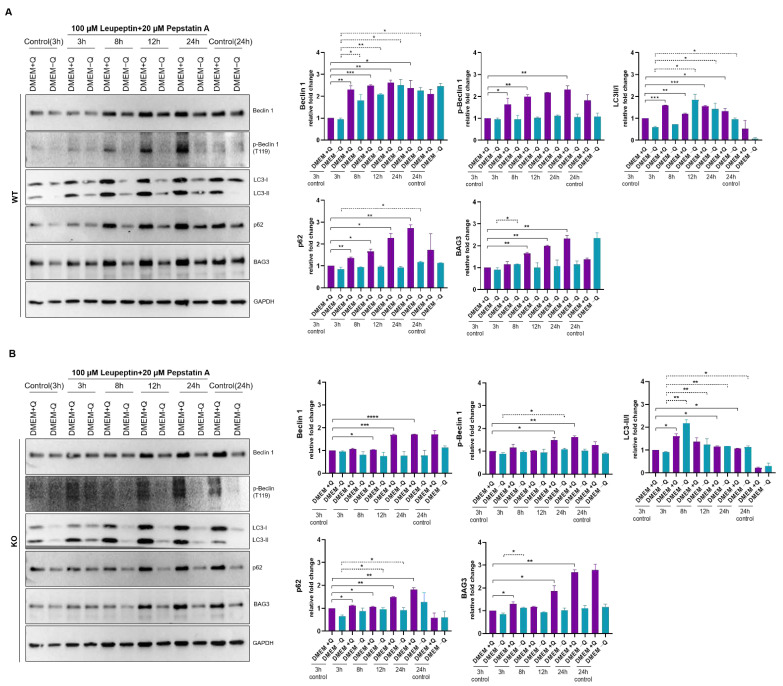
Bag-1 participates in autophagic regulation in breast cancer cells. Immunoblotting of cell lysates from (**A**) wild-type (WT) and (**B**) Bag-1 knock-out (KO) MCF-7 cells that were treated in normal (DMEM + Q) or serum and glutamine-free conditions (DMEM − Q) each containing 100 µM leupeptin and 20 µM pepstatin A for inhibition of lysosomal degradation. Treatments were applied for 3, 8, 12, and 24 h (h). Densitometric analysis was carried out according to GAPDH levels. 20 µg protein samples were loaded to each well in all panels.

Serum and glutamine starvations were previously reported to induce autophagic process in various cell lines [46,47,48,49]. We observed that simultaneous starvations of glutamine and serum caused stronger autophagic response in MCF-7 cells compared to glutamine starvation alone (Figure S3). During autophagy process, LC3 [50] and p62 [50,51] were shown to be degraded by the autophagy machinery. For this reason, levels of LC3 and p62 are in equilibrium in the absence of autophagosomes or lysosomal protease inhibitors (Figure S4), while they accumulate in the presence of such inhibitors. To track the autophagic flux accurately, we treated cells with leupeptin and pepstatin A during starvation. These chemicals were known to inhibit acid proteases of lysosomes, thus they result in accumulation of autophagy proteins upon autophagy induction. Concentrations of inhibitors were determined based on literature [52,53]. According to our data, levels of both LC3-I and LC3-II increased markedly upon autophagy induction in both cell lines under normal media conditions. This data indicates that treatment with leupeptin and pepstatin was successful for lysosomal inhibition. LC3-II/I ratio increased significantly upon 3 h, 8 h and 12 h leupeptin and pepstatin A treatment in cells grown in DMEM + Q. On the other hand, LC3-II/I ratio increased in wild-type cells upon 8 h, 12 h or 24 h treatment in DMEM − Q (Figure 7A), while LC3-II/I ratio reached maximum at 3 h in inhibitor-treated Bag-1 knock-out cells grown in DMEM − Q (Figure 7B). In wild-type cells, p62 levels were higher in cells grown in DMEM + Q and treated for 8 h, 12 h and 24 h with inhibitors compared to 3 h control cells, while such an increase was observed in Bag-1 knock-out cells grown in DMEM + Q and treated for 12 h and 24 h (Figure 7). p62 levels were slightly increased in wild-type MCF-7 cells grown under starvation conditions for 24 h, however, p62 accumulation was observed in 8 h and 12 h inhibitor treated Bag-1 knock out cells upon starvation. Beclin 1 levels significantly increased in wild-type cells independent of starvation. On the other hand, increase in Beclin 1 levels in Bag-1 knock-out cells grown under starvation was slight. Additionally, Beclin 1 levels did not altered in Bag-1 knock-out cells grown under starvation. Wild-type MCF-7 cells resulted in increased phosphorylation level of Beclin 1 under normal conditions, while lack of Bag-1 resulted in weaker response. Besides, p-Beclin 1 levels did not altered in both wild-type and Bag-1 knock-out cells grown under starvation.

In Bag-1 knock-out cells under nutrient-rich conditions, BAG3 levels in the presence of lysosome inhibitors was significantly higher than in their absence. However, the response of wild-type cells to inhibitor treatment was more rapid than that of Bag-1 knock-out cells. Also, starvation induction did not affect BAG3 levels of both cell lines (Figure 7). Densitometric analyses were performed according to their GAPDH levels.

## 3. Discussion

In mammalian cells, protein homeostasis is ensured by chaperone pathways and proteasomal degradation. When the damage has become unresolvable, cells switch from proteasomal degradation to autophagy as another route to cope with systematic overload. The regulation of proteasome/autophagy switch is still largely unknown [3,47]. Here, we suggest that Bag-1 is involved in autophagy regulation through association with Beclin 1.

To investigate probable Bag-1/Beclin 1 interaction, we first performed His_6_-pull down experiments reciprocally in three different breast cancer cell lines, ranging from triple negative to triple positive. These experiments included precipitation of Bag-1S, Bag-1L or Beclin 1 and were performed under normal conditions. In all cell lines tested, Beclin 1 and Bag-1 isoforms were found co-precipitated, which can surprisingly associate Bag-1 to Beclin 1-dependent autophagy. On the other side, our pull-down procedure included ~3h incubation of the cell lysates on the beads. This process may be sufficient for two structurally compatible proteins to bind each other in vitro, even though they do not interact endogenously. Bag-1L isoform is found exclusively in the nucleus while Beclin 1 is mainly localized to cytoplasm, suggesting that these two proteins are unlikely to interact endogenously. However, nuclear localization of Beclin 1 is shown in the literature [54] and this nucleus localized Beclin 1 may harbor endogenous Beclin 1/Bag-1L interaction. However, interaction between Bag-1S and Beclin 1 seemed more probable due to cellular localization of Bag-1S, Beclin 1 and other autophagy machinery members in cytoplasm. To make sure whether Bag-1L and Beclin 1 interaction is misleading and to confirm the cellular localization of Bag-1S and Beclin 1, we studied interaction between Bag-1 isoforms and Beclin 1 in situ. As we considered, Bag-1L and Beclin 1 were localized to different compartments in the cell, while both Bag-1S and Beclin 1 were found co-localized to cytoplasm in all cell lines.

In order to determine whether Bag-1 is a direct or indirect regulator of Beclin 1, Bag-1S and Beclin 1 were affinity-purified and their direct binding ability was analyzed in vitro. Bag-1/Hsp70 interaction was used as positive control. Our results suggested that Bag-1 and Beclin-1 are found within the same protein complex although they do not directly interact with each other. This implies that at least one other linker protein is necessary for Bag-1 to associate with Beclin 1. Bcl-2 seemed the most probable option to us due to its well-known interactions with both Bag-1 and Beclin 1 [48,49]. Therefore, Bag-1 may bind to Beclin 1 through Bcl-2 by forming a tripartite complex. However, unlike previous reports, we observed very weak Bcl-2/Bag-1 interaction in cell lines used in this study. Therefore, we speculate that another protein could mediate the association of Bag-1 and Beclin 1. Alternatively, Bag-1 and Beclin 1 could be involved in one of the many complexes of Bcl-2. This might result in only a small fraction Bcl-2 protein appears to interact with Bag-1 in pull-down experiments, although this amount of interaction could be biologically significant. Thus, to determine whether Bcl-2 plays a role in Beclin 1/Bag-1 interaction for future studies, purified Bcl-2 protein needs to be included in the in vitro binding assays, which we were constraint to do so in this particular study.

To subsequently figure out how Bag-1/Beclin 1 interaction alters autophagy regulation in further steps, we examined the role of p-Bcl-2 (S70) and p-Beclin 1 (T119) phosphorylations [38] on their association with Bag-1. Among the three tested cell lines, BT-474 was the only one in which Bag-1 interacts with p-Beclin 1 (T119) and p-Bcl-2 (S70), which might account for some of the variations cellular responses within these three cell lines. Also, p-Beclin 1 band was observed as ~120 kDa, although molecular weight of Beclin 1 monomer is 60 kDa. Bag-1 probably interacts with Beclin 1 dimer in BT-474 cells.

Further, we sought to determine the formation of Bag-1S/Beclin 1 complex under starvation conditions by precipitating Bag-1S from starved MCF-7 and BT-474 cells. Upon starvation, Bag-1S/Beclin 1 interaction was not altered in MCF-7 cells, but became evident in BT-474 cells. Besides, Bag-1S/p-Beclin 1 interaction was induced in MCF-7 cells, while it was conserved in BT-474 cells under starvation. LC3 was found unbound in MCF-7 cells, while starvation induced Bag-1S/LC3 interaction in BT-474 cells. Overall, these data showed that Bag-1S is involved in the nucleation step of macroautophagy in MCF-7 cells, while it participates to the autophagy in both nucleation and elongation stages in BT-474 cells. These findings together suggest the presence of different autophagy regulation mechanisms upon HER2 upregulation. PIK3CA is known to be mutated in MCF-7 and BT-474 cell lines (E454K and K11N, respectively), which results in AKT activation [54]. Additionally, HER2 overexpressing BT-474 cells, together with PIK3CA mutation, display higher AKT phosphorylation, compared to MCF-7 cells [54]. Also, HER2 overexpressing cell lines were found to show higher p-AKT levels under serum starved conditions, regardless of PIK3CA mutations [55]. AKT kinases (AKT1-3) are localized downstream of PI3Ks and activates ERα [54]. AKT phosphorylates many transcription factors and also controls proliferation and survival pathways through phosphorylation [56]. mTOR is one of the major regulators of autophagy and is a downstream effector of AKT and PI3K [38]. Based on this knowledge, autophagy pathways are differentially regulated in MCF-7 and BT-474 cells and basal activation of AKT in BT-474 cells may result in higher basal autophagic activity.

In macroautophagy, after Beclin 1 is dissociated from Beclin 1/Bcl-2 complex, pre-autophagosomal complex is formed, which is necessary for autophagy initiation [26,27,50,51]. Apoptotic cell death and autophagic cell survival mechanisms are intricately controlled and can happen simultaneously or, apoptosis can be inhibited by induction of autophagy or vice versa [52,53]. Bcl-2/Beclin 1 complex was found to be easily dissociated after a short period of nutrient deprivation (4 h), whereas dissociation of Bcl-2/Bax complex require a longer period of nutrient deprivation (16 h) [37]. Rapid dissociation of Bcl-2/Beclin 1 complex may occur to initiate cell survival process as fast as possible [37]. Besides, this mechanism is an example of the regulation of apoptosis and autophagy by the same intermediates by differential binding affinities. Unlike other BH3-only proteins, which carries a hydrophobic residue at that position, Beclin 1 has a Thr at that specific position allowing it to be easily released from the complex [38]. Our results indicated that Bag-1/Beclin 1 interaction occurs before Beclin 1 is phosphorylated and dissociated from Bcl-2.

Bag-1S/Beclin 1 interaction was observed in both MCF-7 (Figure 4A) and BT-474 (Figure 4F) cells independent of media condition. Nevertheless, this interaction was stronger under autophagic conditions in BT-474 cells. In MCF-7 cells, Bag-1 does not associate with p-Beclin 1 (T119) as effectively in standard media conditions. On the other hand, Bag-1/p-Beclin 1 (T119) interaction was reinforced during nutrient starvation (Figure 4B). Consistently, Beclin 1 levels were reduced throughout starvation in Bag-1 KO cells, probably suggesting that Bag-1 might stabilize Beclin 1 during starvation. On the other side, Bag-1S and p-Beclin 1 (T119) were observed together in BT-474 samples independent of autophagy induction. Additionally, p-Beclin 1 was observed as higher molecular weight protein in Bag-1S overexpressed samples compared to samples collected from untransfected cells. Also, in contrast to MCF-7 cells, p-Beclin 1 was observed as double band in BT-474 cells. This increase and double band formation may arise from post-translationally addition of molecular tag(s), which probably stabilize phosphorylated form of Beclin 1. Through this mechanism, durability and persistence of autophagy might be provided (Figure 4F). This mechanism may increase survival chance of BT-474 cells. Although mechanistical role of Bag-1 within Bag-1/Beclin 1 complex is not fully understood, our results might argue that Bag-1 functions to protect Beclin 1 from proteasomal or autophagosomal degradation, especially during stress conditions such as nutrient starvation. This mechanism might be important for sustaining autophagy as cleavage of Beclin 1 was shown to induce apoptosis in different contexts [54,57].

LC3 lipidation (turnover of LC3-I to LC3-II) is an essential step in autophagosome formation, therefore LC3-II/I ratio is often used as an indicator of autophagic flux. In addition, adaptor protein p62/SQSTM1 recruits ubiquitylated proteins to the phagophore and works with LC3-II during autophagy. Therefore, increase in LC3-II/I ratio and decrease in p62 levels are often associated with activated macroautophagy [40,41]. However, these proteins are localized to autophagosome and are degraded by autophagosomal machinery in the absence of autophagosome or lysosome inhibitors [50,51]. Therefore, autophagic flux is more accurately represented in the presence of autophagosome or lysosomal protease inhibitors.

We further investigated the role of Bag-1 on autophagic regulation in both wild-type and Bag-1 knock out MCF-7 cells under nutrient-rich (DMEM + Q) or −deprived (DMEM − Q) conditions. According to our data, the lack of Bag-1 retarded autophagy induction in knock-out cells grown under nutrient rich conditions and response was observed later compared to wild-type cells, which indicates lower basal autophagy levels of Bag-1 knock-out cells under nutrient-rich conditions. On the other hand, neither of cells responded serum- and glutamine deprivation through increasing levels of autophagy markers. Probably effect of both serum- and glutamine-deprivation was fatal enough to prevent autophagy induction.

BAG3, together with HSP70, HSPB8 and p62, is responsible for selective degradation of aggregation-prone proteins through autophagy (also known as BAG3-mediated selective macroautophagy or CASA) [9]. Since proteasomal degradation/autophagy switch is instructed by cellular Bag-1/BAG3 ratio [3], we expected the accumulation of BAG3 in Bag-1 knock-out cells upon nutrient deprivation to compensate lack of Bag-1. However, neither wild-type nor knock-out cells responded serum- and glutamine-deprivation through BAG3 accumulation over time, showing their inability to initiate BAG3-mediated autophagy under this condition. According to literature, activity of autophagy pathway is upregulated in response to impaired proteasome activity to maintain cellular homeostasis [3], but data are scarce on reverse regulatory mechanism [58]. On the other hand, reduced autophagy mediated by lysosomal inhibition may result in reduced proteasomal activity [58]. From that point of view, lysosomal protease inhibitors used for the inhibition of autophagy may also lead to improper proteasomal activity. Under these circumstances, cells may attempt to compensate inhibition of both BAG3-mediated autophagy and proteasome pathways through activating Beclin 1-dependent autophagy or other autophagy pathways. This may explain the sharp increase in Beclin 1 levels in wild-type cells in the presence of inhibitors. The increase in Beclin 1 levels were not that high in Bag-1 knock-out cells, which might be explained by the effect of Bag-1 on Beclin 1 levels. In wild-type MCF-7 cells, both BAG3 levels and LC3 lipidation were found higher in cells grown under nutrient-rich conditions. Although mechanism is still not known, BAG3 is known to protect levels of total LC3 protein through controlling its translation and this effect was shown to be specific only to LC3, not to other ATGs [59]. Higher LC3 levels in wild-type cells, which harbor higher BAG3 levels can be explained by this role of BAG3 on LC3. On the other hand, in both trypan blue staining and MTT experiments, Bag-1 knock-out cells resulted in reduced cell viability and proliferation under both normal and starvation conditions compared to wild-type cells. Because Bag-1 knock-out cells exhibited reduced levels of BAG3 compared to wild-type cells, these cells harbor reduced levels of both Bag-1 and BAG3 than their normal cellular levels. Bag-1 and BAG3 co-silencing experiment performed in HL-60 cells harmed cell survival and induced cytochrome C release and cell death through apoptosis. Also, p-ERK1/2 levels were reduced in these co-silenced cells [60]. According to this data, dramatically reduced cell survival in Bag-1 knock-out cells may result from reduced levels of BAG3 in addition to lack of Bag-1.

In conclusion, our study demonstrates that Bag-1 plays a role in controlling cell survival/cell death decision through indirectly interacting with Beclin 1. Although our study was limited to determine the mechanism behind Bag-1/Beclin 1 interaction and the precise function of Bag-1 on Beclin 1-mediated autophagy, our findings highlight a potential role of Bag-1 in macroautophagy regulation.

## 4. Materials and Methods

### 4.1. DNA Constructs and siRNAs

Full-length Bag-1L (N-His_6_BAG-1L), Bag-1S (N-His_6_Bag-1S) and Beclin 1 (N-His_6_Beclin 1) containing an N-terminal hexahistidine (His6) tag with a tobacco etch virus (TEV) protease cleavage site were amplified by PCR, cloned into pcDNA 3.1 mammalian expression vector through ligation-independent cloning, and sequenced.

### 4.2. Antibodies

Monoclonal antibodies used in the study were mouse α-BAG-1 (3920), rabbit α-LC3-I/II (2775), rabbit α-Beclin 1 (3495), rabbit α-p62 (5114), rabbit α-GAPDH (5174). All primary antibodies (1:500) and HRP-conjugated anti-rabbit (5615) or anti-mouse (7076) secondary antibodies (1:3000) were purchased from Cell Signaling Technologies (Danvers, MA, USA). Rabbit α-p-Beclin 1 (T119) (ABC118) antibody was purchased from Sigma-Aldrich (Steinheim, Germany).

### 4.3. Cell Lines, Cell Culture and Transient Transfection

MDA-MB-231 (ATCC^®^ HTB-26™), MCF-7 (ATCC^®^ HTB-22™), BT-474 (ATCC^®^ HTB-20™) breast cancer cells and HEK293T (ATCC^®^ CRL-3216™) cells were cultured in high glucose DMEM medium (Dulbecco’s Modified Eagle’s medium, 41966029, Gibco, Wlatham, MA, USA) supplemented with 10% fetal bovine serum (10500064, Thermo, Waltham, MA, USA) and 100 U/100 mg mL^−1^ penicillin/streptomycin (15140122, Gibco) in 5% CO_2_ humidified air at 37 °C. 5 × 10^5^/well MDA-MB-231, MCF-7 or BT-474 cells were seeded into 6-well plates. Upon reaching 80% confluency, cells were transfected with 1 µg N-His_6_Bag-1S, N-His_6_BAG-1L or N-His_6_Beclin 1 plasmid diluted in 100 µL serum-free DMEM medium using PEI 25K in vitro transfection reagent (23966-1, Polysciences, Warrington, PA, USA) with a 1:3 ratio, according to the manufacturer’s instructions.

### 4.4. CRISPR/Cas9-Mediated Bag-1 Knockout

Bag-1 knock-out MCF-7 cells were generated using CRISPR/Cas9 system as described previously [61,62]. Briefly, Bag-1 CRISPR/Cas9 Knockout plasmid (sc-417179), Bag-1 Homology Directed Repair plasmid (sc-417179-HDR) and Control CRISPR/Cas9 Knockout plasmid (sc-418922) was purchased from Santa Cruz Biotechnology (California, CA, USA). Following the transfection with CRISPR/Cas9 plasmids, single cell selection and colony screening were performed. Bag-1 knockouts were validated by immunoblotting.

### 4.5. Proliferation/Survival Rates

Proliferation rates were determined by cell counting assays as described previously [63]. Briefly, 100,000 cell/well WT and 150,000 cell/well Bag1-KO MCF-7 cells were seeded into 6-well plates. After adhering overnight, initial cell number was determined from reference plates following trypsinization. Then the experimental wells were washed with PBS and media conditions (full-DMEM or DMEM without glutamine) were applied. Final cell numbers were counted 3 days after the experiments using Countess cell counter (Invitrogen). Percent of alive cells were determined by trypan blue staining. Proliferation/survival rate was calculated by the following formula:

$$Proliferation\;Rate\;\left(\frac{Doublings}{Day}\right)=\frac{log2\left(\frac{Final\;Cell\;Count}{Initial\;Cell\;Count}\right)}{Day}$$

Proliferation assays for Bag-1S and Beclin 1 overexpressing cells were performed following transient transfection. MCF-7 cells were transfected with empty pcDNA3.1 vector, N-His_6_Bag-1S or N-His_6_Beclin 1 as described above. Eight h after transfection, 100,000 cells/well was seeded into 6-well plates and proliferation rate was determined as described above.

### 4.6. MTT Cell Viability Assay

MCF-7 wild-type and Bag-1 knock-out cells were seeded at 8 × 10^3^ cells/well in 4-replicate in 96-well plates and cultured in high glucose DMEM media containing serum and glutamine. The day after, media was removed and wells were washed with PBS. WT and KO cells were grown in serum and glutamine containing (shown as DMEM + Q) or serum and glutamine-free media (shown as DMEM − Q) for 24, 48 or 72 h. 10 µl MTT reagent (3-(4,5-dimethylthiazol-2-yl)-2,5-diphenyltetrazolium bromide) was added to each well and cells were incubated for 3 h at 37 °C. Media was then removed and formed formazan crystals were dissolved by 100 µL dimethyl sulfoxide (DMSO). Cell viability was quantified by reading absorbance at 570 nm using a microplate reader.

### 4.7. Starvation

MCF-7 cells were cultured under serum (0.1%) and glutamine (0%) starvation for 3 to 24 h to induce autophagy. Total protein was extracted from serum-starved cells. Levels of Bag-1 and Bag-3, also autophagy markers Beclin 1, p-Beclin 1 (T119), LC3 and p62 were analyzed by western blotting. Transient Bag-1 transfection was performed as described. Starvation was performed in Bag-1 overexpressed cells post transfection. To monitor autophagic flux within the given time period, 100µM leupeptin and 20 µM pepstatin A were added to starvation media to block lysosomal degradation.

### 4.8. Immunoblotting

Cells were detached by manual scraping, collected by centrifugation and lysed in NP-40 cell lysis buffer (150 mM NaCl, 50 mM Tris, 1% NP-40) supplemented with protease and phosphatase inhibitors. Lysates were centrifuged at max speed for 15 min to remove cell debris. Protein concentrations were determined by Bradford assay (Bio-Rad, Richmond, CA, USA) using bovine serum albumin (BSA; 2 mg/mL) as a standard. The quantified protein samples were stored at −20 °C for further use. 20 µg protein lysate was run on 12% polyacrylamide gel. Blotting was performed by using 0.45 µm Nitrocellulose membrane (Bio-Rad) and wet transfer system (GE TE 22 Mini Tank Transfer Unit) overnight at 4 °C and 16 V. Membranes were blocked with 5% non-fat dry milk in TBS-T (Bio-Rad). Primary antibody binding was performed overnight at 4 °C with gentle agitation. Membranes were washed with TBS-T and incubated with secondary antibody at RT for 2 h. After the final wash, membrane was treated with Clarity™ ECL Western Blotting Substrate (Bio-Rad) and visualized via ChemiDoc Imaging System (Bio-Rad, Richmond, CA, USA).

### 4.9. His-Pull Down

Forty eight h after His_6_Bag-1S, His_6_Bag-1L or His_6_Beclin 1 transfections, cells were lysed with NP-40 buffer supplemented with protease and phosphatase inhibitors. Lysates were subjected to Ni-NTA affinity isolation under native conditions. Appropriate amount of Ni-NTA agarose beads were incubated with approximately 1 mg cell lysate (supplemented with 20 mM imidazole) for 2.5 h with gentle rotation at 4 °C. Bead-protein complexes were washed mildly for three times with 20 mM imidazole in wash buffer (100 mM Hepes, 150 mM NaCl, pH 7.4). Protein complexes were eluted with 500 mM imidazole in wash buffer. Fractions were analyzed by immunoblotting. Only bead and untransfected cell lysates were used as negative controls. For pull-down experiments under starvation, glutamine and serum withdrawal were also applied 48 h after transfections.

### 4.10. Immunocytochemistry

Cells were seeded as 1 × 10^5^ cells per well in 12-well plate containing poly-L-lysine coated coverslips, and co-transfected with His_6_Bag-1S or His_6_Bag-1L and His_6_Beclin 1 plasmid. 48 h post-transfection, culture medium was discarded, and cells were washed with phosphate buffered saline (PBS) solution. Cells were fixed in pre-chilled 4% paraformaldehyde and incubated for 10 min at room temperature, then, washed three times with PBS. Cells were blocked by 1 h incubation in blocking solution (3% BSA, 0.1% Triton-X 100 in PBS) and were incubated with appropriate primary antibodies overnight at 4 °C. Primary antibodies used were mouse anti-Bag-1 (1:200), rabbit anti-Beclin 1 (1:200). Following washing, cells were incubated for 1 h at RT with the secondary antibody (Alexa Flour^®^ 488 goat anti-mouse or Alexa Flour^®^ 594 goat anti-rabbit; 1:300 for both, Invitrogen, Carlsbad, CA, USA). After extensive washing, coverslips were mounted on slides using ProLong Diamond Antifade mounting medium containing DAPI (P36962, Thermo Fisher, Waltheim, MA, USA). Confocal images were obtained via a Leica TCS SP2 SE confocal microscope (Leica, Buffalo Grove, IL, USA).

### 4.11. In Vitro Binding Assay

His_6_Bag-1S overexpressing HEK293T cells were detached by manual scraping, collected by centrifugation and lysed in RIPA cell lysis buffer (Santa Cruz) supplemented with protease and phosphatase inhibitors. Lysates were centrifuged at max speed for 15 min to remove cell debris. First Ni-NTA purification procedure was carried out to isolate His_6_Bag-1S from the protein mixture. Protein sample was incubated with Ni-NTA agarose beads at 4 °C, for 4 h. Flow-through was aspirated and resin particles were washed away to eliminate weakly bound impurities using high-salt buffer (20 mM sodium phosphate, 500 mM NaCl, 20 mM imidazole, 0.5% Triton-X 100, 10% glycerol, pH 7.8) and ATP/MgCl_2_/KCl containing wash buffer [64]. His_6_Bag-1S was recovered by elution with imidazole. Following the first purification step, the eluate containing His_6_Bag-1S fusion protein was subjected to TEV protease cleavage (Actev, Sigma, Steinheim, Germany) to remove hexahistidine tag. Bag-1S was separated from his-tagged TEV enzyme and impurities with a second Ni-NTA purification step in flow-through mode. After each step, samples were monitored by immunoblotting and SDS-PAGE. Purified his-tagged proteins were dialyzed into equilibration buffer (20 mM HEPES buffer, 150 mM NaCl, pH 7.4). His_6_Beclin 1 containing lysates were incubated with Ni-NTA agarose beads at 4 °C, for 4 h. Beads were washed four times with wash buffer (100 mM sodium phosphate, 500 mM NaCl, 20 mM imidazole, 10% glycerol, 10 mM beta-mercaptoethanol, 0.5% Triton X-100, pH 7.8). Attached His_6_Beclin 1 was incubated with 20 µg purified untagged Bag-1S at 37 °C for 30 min. Beads were washed with a mild wash buffer (20 mM HEPES, 150 mM NaCl, 5 mM MgCl_2_, pH 7.4). Finally, proteins were eluted with 500 mM imidazole in wash buffer and monitored by immunoblotting. While Bag-1 only and Beclin 1 only samples were used as negative controls, Bag-1S/Hsp70 interaction was included as a positive control. To test the binding of Bag-1S/HSP70 in vitro, His_6_Bag-1S was first captured by Ni-NTA beads and stringently washed with high-salt buffer and ATP/MgCl_2_/KCl containing wash buffer as described above. Immobilized Bag-1S was incubated with purified Hsp70 (20 µg in 20 mM HEPES, 5 mM MgCl_2_ and 150 mM KCl, pH 7.4) for 30 min at 30 °C. Bag-1S/Hsp70 complexes were eluted with 500 mM imidazole in wash buffer and fractions were then subjected to western blot analysis.

### 4.12. Statistical Analysis

All experiments were performed using at least three independent replicates (i.e., different passages from each cell lines). Single comparison between two groups, is calculated via two-tailed, unpaired student’s *t*-test. Significance levels: * *p* ≤ 0.05, ** *p* ≤ 0.01, *** *p* ≤ 0.001.

## Data Availability

The data presented in this study are available on request from the corresponding author.

## Supplementary Materials

The following are available online. Figure S1: Beclin 1 interacts with both Bag-1S and Bag-1L isoforms. Figure S2: Interaction of Bag-1S and Beclin 1 is revealed in situ by immunocytochemistry assay. Figure S3: Bag-1 participates in autophagic regulation in breast cancer cells. Figure S4: Bag-1 participates in autophagic regulation in breast cancer cells.

## Sample Availability

Sample of compounds are not available from the authors.
