# Supplementary material for: Co-Chaperone Bag-1 Plays a Role in the Autophagy-Dependent Cell Survival through Beclin 1 Interaction
Source: Molecules. 2021 Feb 6;26(4):854. doi: 10.3390/molecules26040854 (PMC7914623; doi:10.3390/molecules26040854)
Supplement: Supplementary file 1 [file molecules-26-00854-s001.pdf]

molecules

Supplementary Material

Co-Chaperone Bag-1 Plays a Role in the Autophagy-Dependent Cell Survival Through Beclin-1 Interaction

Miray Turk [1,†], Ozge Tatli [1,2,†], H. Furkan Alkan [3], Pelin Ozfiliz Kilbas [4], Gizem Alkurt [1] and Gizem Dinler Doganay [1,3,*]

[1] Molecular Biology, Genetics-Biotechnology, Graduate School of Science, Engineering and Technology, Istanbul Technical University, Istanbul, Turkey
[2] Department of Molecular Biology and Genetics, Istanbul Medeniyet University, Istanbul, Turkey
[3] Department of Molecular Biology and Genetics, Istanbul Technical University, Istanbul, Turkey
[4] Department of Molecular Biology and Genetics, Istanbul Kultur University, Istanbul, Turkey
† These authors contributed equally to this work.
* Correspondence: gddoganay@itu.edu.tr

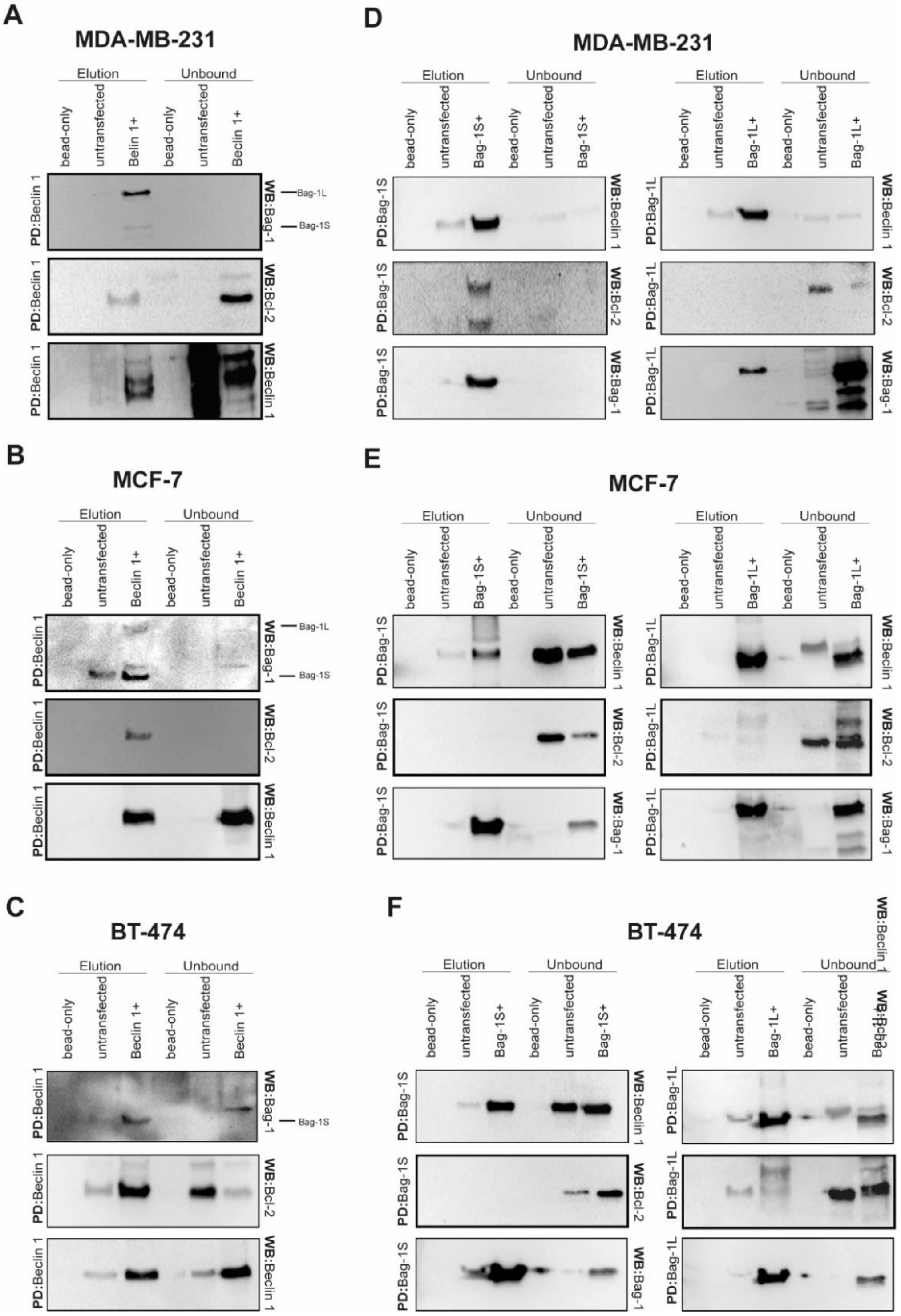

Figure S1. Beclin 1 interacts with both Bag-1S and Bag-1L isoforms. A-C) His_6Beclin 1 was his-pulled down by Ni-NTA beads from (A) MDA-MB-231, (B) MCF-7, and (C) BT-474 cell lysates. After wash, bound Beclin 1-related complexes were released by imidazole. As a control, precipitations were also carried out in parallel experiments with untransfected cell lysate and empty Ni-NTA beads. Elution and unbound fractions were subjected to western blot analysis using anti-Bag-1, anti-Bcl-2, anti- Beclin 1 antibodies. D-F) Both His_6Bag-1S and His_6Bag-1L were his-pulled down in (D) MDA-MB-231, (E) MCF-7 and (F) BT-474 cells as reciprocal experiments and analyzed by western blotting. Bag-1 and Beclin 1 were co-precipitated in both directions.

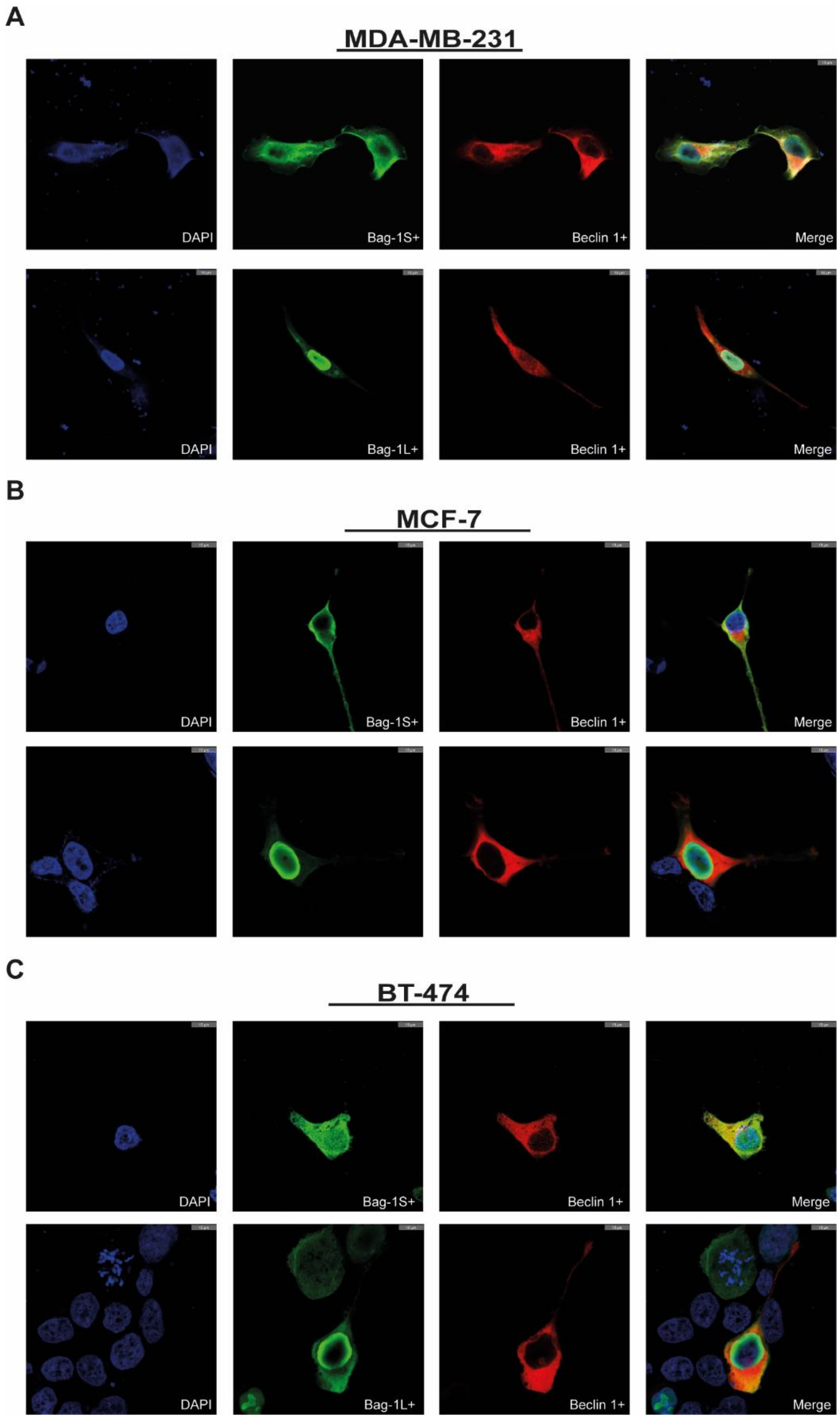

Figure S2. Interaction of Bag-1S and Beclin 1 is revealed in situ by immunocytochemistry assay. Interaction of Bag-1S and Beclin 1 is revealed in situ by immunocytochemistry assay. (A) MDA-MB-231, (B) MCF-7, and (C) BT-474 cells were co-transfected with His$_6$Beclin 1 and His$_6$Bag-1S or His$_6$Bag-1L and their co-localization is investigated via immunocytochemistry. Bag-1S and Bag-1L is independently stained green and Beclin 1 was stained as red while nucleus was shown in blue. Co-localized areas were represented in yellow. Scale bar equals to 10μm. In all cell lines, Bag-1S and Beclin 1 was observed in cytoplasm while Bag-1L is located inner side of nuclear membrane. Only small isoform of Bag-1 is observed co-localized to Beclin 1.

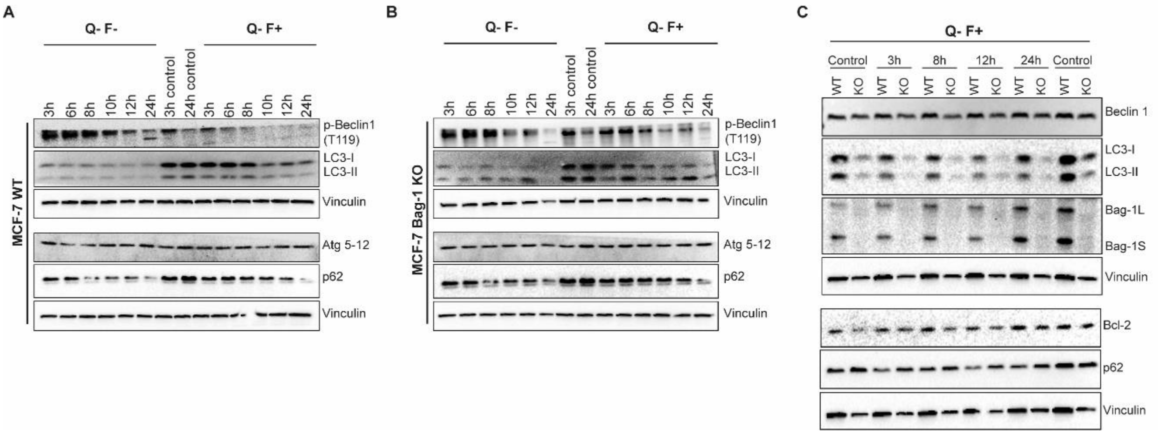

Figure S3. Bag-1 participates in autophagic regulation in breast cancer cells. (A,B) Immunoblotting of cell lysates from wild-type (WT) (A) and Bag-1 knock-out (KO) (B) MCF-7 cells that were treated in only glutamine-free (Q-F+) or serum and glutamine-free (Q-F-) conditions for 3, 6, 8, 10, 12, and 24 hours (h). Controls include lysates from cells treated with standard DMEM for 3 (left) and 24 hours (right). Expression of some autophagy markers including p-Beclin 1 (T119), LC3, p62, and Atg5-12 were detected. Vinculin was used as endogenous control. (C) Immunoblotting of cell lysates from wild-type (WT) and Bag-1 knock-out (KO) MCF-7 cells treated in glutamine-free conditions for 3, 8, 12 and 24 hours. Controls include lysates from cells treated with standard DMEM for 3 hours (left) and 24 hours (right). Bag-1 expression together with some autophagy markers including Beclin 1, LC3, p62, and Bcl-2 were detected. GAPDH was used as endogenous control. 20 µg protein samples were loaded to each well in both panels.

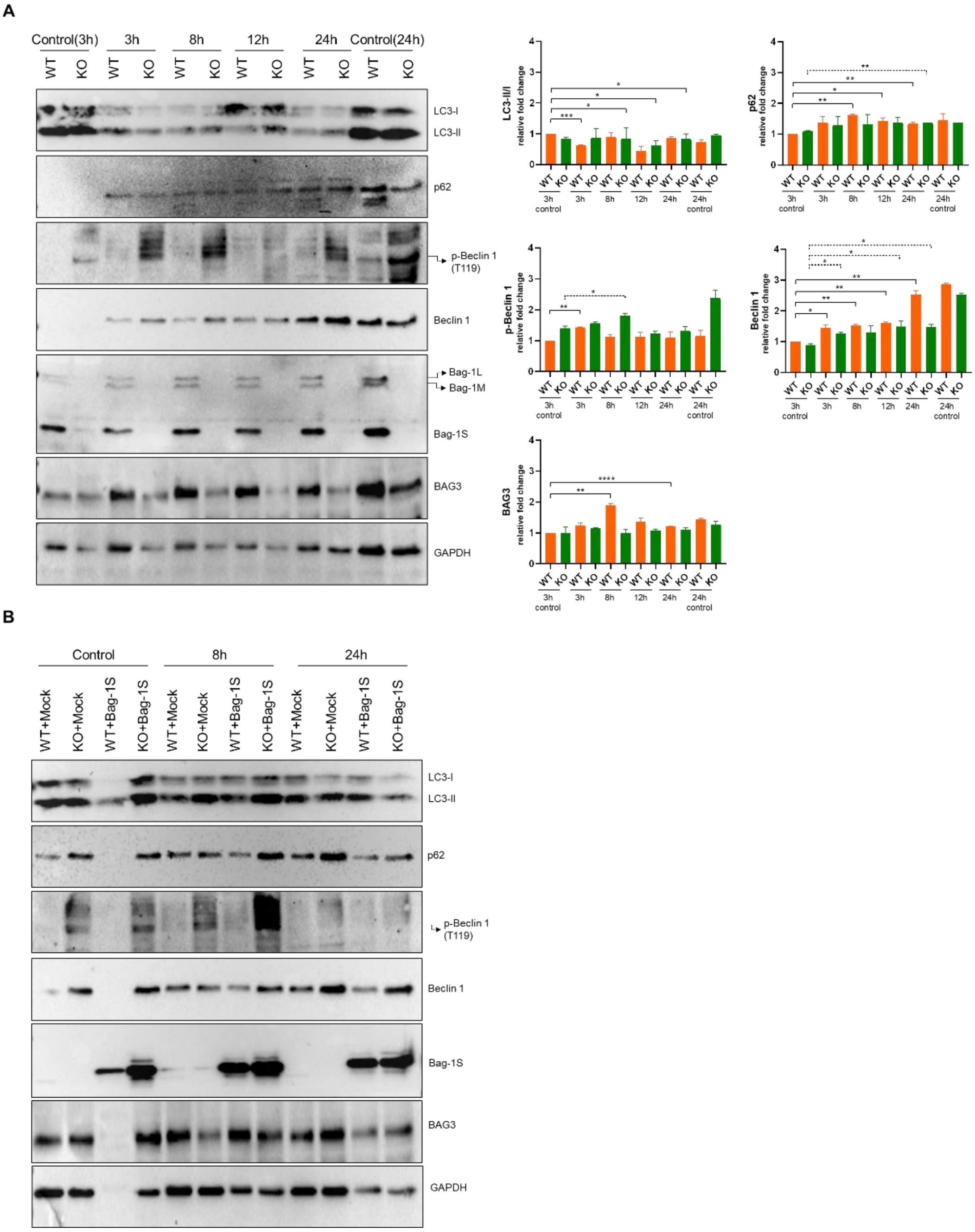

Figure S4. Bag-1 participates in autophagic regulation in breast cancer cells. (A) Immunoblotting of cell lysates from wild-type (WT) and Bag-1 knock-out (KO) MCF-7 cells that were treated in serum and glutamine-free conditions for 3, 8, 12, and 24 hours (h). Controls include lysates from cells treated with standard DMEM for 3 (left) and 24 hours (right). Bag-1 expression together with some autophagy markers including LC3, p62, p-Beclin 1 (T119), and Beclin 1 were detected. BAG3 levels were also measured to screen Bag-1/BAG3 ratio. GAPDH was used as endogenous control. (B) Immunoblotting of cell lysates from wild-type (WT) and Bag-1 knock-out (KO) MCF-7 cells that were transfected with *Mock* or *Bag-1S* vectors and treated in serum-free and glutamine-free conditions for 8 and 24 hours. Controls include lysates from cells treated with standard DMEM for 24 hours. Bag-1 expression together with some autophagy markers including LC3, p62, p-Beclin 1 (T119), Beclin 1 were detected. BAG3 levels were also measured to screen Bag-1/BAG3 ratio. GAPDH was used as endogenous control. 20 μg protein samples were loaded to each well in both panels.
